# The Effects of Digital Interventions on Language Development in Children with Autism Spectrum Disorder: A Systematic Review and Interdisciplinary Synthesis

**DOI:** 10.3390/pediatric18040090

**Published:** 2026-07-08

**Authors:** Murat Demirekin, Hatice Yalçın

**Affiliations:** 1 Department of English Language Teaching, Faculty of Education, Selcuk University, Konya 42400, Turkey; murat.demirekin@selcuk.edu.tr; 2 Department of Child Development Special Education, Faculty of Health Science, Cyprus Health and Social Science University, Güzelyurt 99440, Cyprus

**Keywords:** Autism spectrum disorder, video modeling, game-based applications, artificial intelligence-supported systems, systematic review

## Abstract

**Background/Objectives:** Digital technologies are increasingly used in interventions for children with Autism Spectrum Disorder (ASD) to support language development. However, existing evidence remains fragmented due to heterogeneity in intervention types, participant characteristics, and outcome measures. This systematic review aims to synthesize current empirical findings on the effects of digital interventions on language development in children with ASD and to identify key factors influencing intervention effectiveness. **Methods:** A systematic review was conducted in accordance with PRISMA 2020 guidelines. Searches were performed in PubMed, Scopus, Web of Science, ERIC, and PsycINFO for studies published between 2010 and 2025. Eligible studies included experimental, quasi-experimental, and intervention-based designs involving children aged 2–18 years with ASD and reporting at least one language-related outcome. Data extraction was performed independently by two reviewers using a structured form. Methodological quality was assessed using the Joanna Briggs Institute (JBI) checklist and CASP tools. Due to heterogeneity across studies, a narrative synthesis approach was applied. **Results:** A total of 61 studies met the inclusion criteria. Findings indicate that digital interventions generally have positive effects on language development in children with ASD, with stronger and more consistent outcomes in receptive and expressive language domains. Intervention effectiveness varied according to duration, intensity, content quality, and contextual factors such as family involvement and technological access. **Conclusions:** The evidence suggests that digital interventions may have positive effects on language development in children with ASD, particularly in receptive and expressive language domains. Among intervention types, video modeling and AI-supported approaches appear to show promising outcomes; however, these findings should be interpreted with caution due to the limited number of AI-focused studies and substantial heterogeneity in study designs, sample characteristics, and outcome measures. Gamified and mobile applications demonstrate moderate effects, especially in vocabulary and pragmatic language skills. Overall, intervention effectiveness varies according to duration, intensity, content quality, and contextual factors such as family involvement and technological access. Future research should prioritize standardized methodologies and longitudinal designs.

## 1. Introduction

Autism Spectrum Disorder (ASD) is a neurodevelopmental condition characterized by persistent challenges in social communication and restricted or repetitive behavioral patterns, with considerable heterogeneity across individuals [1]. One of the most prominent dimensions of this heterogeneity concerns language development [2]. Receptive and expressive language abilities in children with ASD show substantial variability; while some individuals develop fluent speech, others exhibit minimal or limited verbal communication [3]. Pragmatic language use, particularly the functional use of language in social contexts, is frequently reported as a core area of difficulty [4]. This variability highlights the need for individualized and adaptive intervention approaches, increasing interest in technology-supported instructional strategies [5].

In this context, digital technologies have increasingly been integrated into intervention practices. Tablet-based applications, augmented and virtual reality environments, artificial intelligence-supported systems, and video modeling tools may enhance the personalization and repeatability of learning experiences, while also increasing learner engagement [6]. Given that many children with ASD demonstrate preferences for structured and visually supported learning environments, digital tools may offer potentially effective instructional alternatives [7].

In the context of this review, the term “digital intervention” refers to technology-mediated intervention approaches designed to support language development outcomes in children with ASD. These interventions include artificial intelligence-supported systems, video modeling platforms, computer-assisted instructional programs, tablet/mobile applications, gamified learning environments, virtual communication tools, and other interactive digital systems used with therapeutic or educational purposes [2,4]. Although these technologies differ in complexity and delivery mechanisms, they share the common characteristic of utilizing digital interfaces to facilitate language-related learning processes. To reduce conceptual ambiguity, intervention types were categorized according to their technological structure and pedagogical function during the synthesis process [7].

Video modeling has been widely investigated as an evidence-informed approach for teaching communication and behavioral skills in ASD [8]. Visual and step-by-step presentation of behaviors may facilitate language acquisition through modeling processes [2,8]. Similarly, gamified applications may increase engagement through interactive repetition, while AI-supported platforms can provide adaptive feedback based on learner performance [6,9]. Despite these advantages, language development in ASD is a multidimensional construct that extends beyond verbal output and includes phonological, lexical, syntactic, and pragmatic domains [10].

Research indicates that children with Autism Spectrum Disorder (ASD) often experience difficulties in joint attention, social referencing, and interpreting nonverbal cues, which may weaken the cognitive foundations of language acquisition [2,11]. Consequently, processes such as inferring word meaning from context, maintaining conversational flow, and appropriately sequencing speech may deviate from typical developmental trajectories [12].

These challenges suggest that language instruction should not rely solely on verbal input, but may benefit from multimodal approaches incorporating visual supports, structured instructional sequences, and motivational elements [13,14,15]. In this regard, digital tools may offer potential advantages by addressing multiple components of language learning through adaptive and interactive features.

This review was conceptually informed by developmental, behavioral, and socio-cognitive perspectives on language acquisition in children with ASD. From a developmental perspective, digital interventions may support language growth through repeated exposure, multimodal stimulation, and individualized pacing. Behavioral approaches emphasize reinforcement, modeling, and structured learning opportunities frequently embedded within digital systems. In addition, socio-cognitive perspectives highlight the importance of interaction, attention, communication, and contextual engagement in language learning processes [13].

The recent literature suggests that findings regarding the effectiveness of digital interventions for language development in children with ASD remain fragmented. Studies differ in terms of technologies used, participant characteristics, and targeted language outcomes, which limits the comparability of results. Despite the rapid growth of the field, a comprehensive synthesis of the evidence remains limited.

Previous systematic reviews and narrative synthesis examining technology-supported interventions for children with ASD have primarily focused on specific intervention modalities, such as augmentative communication systems, video modeling, or computer-assisted instruction. In addition, many earlier reviews emphasized broader developmental or behavioral outcomes rather than language-specific dimensions. Existing syntheses also show substantial variation in methodological scope, participant characteristics, intervention classifications, and outcome assessment approaches. The present review extends the current literature by providing an interdisciplinary and language-focused synthesis of diverse digital intervention approaches published between 2010 and 2025. Unlike previous reviews that concentrated on isolated technologies or narrow developmental targets, this study comparatively evaluates multiple categories of digital interventions and examines contextual factors influencing language-related outcomes, including intervention intensity, technological structure, family involvement, and accessibility conditions.

Therefore, a systematic evaluation is needed to examine how, under what conditions, and to what extent digital interventions support language development in children with ASD. This systematic review aims to synthesize the existing literature within a unified framework, identify common mechanisms underlying effective interventions, and provide a forward-looking perspective for future research and practice.

The review question was structured according to the PICO framework as follows:

Population (P): Children and adolescents diagnosed with ASD. Intervention (I): Digital technology-based language interventions, including AI-supported systems, video modeling, mobile applications, and computer-assisted instructional tools. Comparison (C): Conventional interventions, control conditions, or pre–post intervention comparisons.

Outcomes (O): Language development outcomes, including receptive language, expressive language, vocabulary acquisition, pragmatic communication, and related linguistic skills. Accordingly, this review aimed to address the following research questions:

1. What are the effects of digital interventions on language development outcomes in children with ASD?
2. Which types of digital interventions demonstrate the most consistent language-related benefits?
3. What contextual and methodological factors influence intervention effectiveness?

## 2. Materials and Methods

### 2.1. Work Design

This study is a systematic review designed to evaluate the effects of digital interventions on language outcomes in children with Autism Spectrum Disorder (ASD). The review was conducted in accordance with the PRISMA 2020 reporting guidelines to ensure methodological transparency and reproducibility [16]. Experimental, quasi-experimental, and intervention-based studies were included. The PRISMA 2020 Checklist is provided as Supplementary Materials (Table S1).

Systematic literature searches were conducted in the Web of Science, Scopus, PubMed, and ERIC databases using predefined keyword combinations. No language restrictions were applied during the search process. The study selection process was carried out in two stages: first, titles and abstracts were screened according to eligibility criteria; second, full-text articles were assessed for inclusion.

In order to increase transparency and traceability of findings, each included study was systematically coded according to intervention type, participant age group, and intervention intensity, and these codes were used to directly link individual studies to thematic synthesis categories.

Data extraction was performed independently by two researchers using a structured extraction form. The form included study design, participant characteristics (age, gender, and diagnostic criteria), intervention characteristics (type, duration, and intensity), comparison conditions, measurement instruments, and key findings. Prior to full data extraction, the form was piloted and refined to ensure consistency.

Methodological quality was assessed using the Joanna Briggs Institute (JBI) critical appraisal tools for experimental and quasi-experimental studies [17]. For qualitative and mixed-methods studies, the Critical Appraisal Skills Programme (CASP) checklists were applied to evaluate methodological rigor and reporting quality [18].

Where available, standardized effect size estimates reported in the included studies were extracted and tabulated descriptively. Due to heterogeneity in outcome measures, intervention designs, and statistical reporting practices, these effect sizes were not statistically pooled. Instead, a structured narrative synthesis approach was used to interpret the range and distribution of reported effects.

Methodological quality was independently assessed by two reviewers using the Joanna Briggs Institute (JBI) critical appraisal checklists and the Critical Appraisal Skills Programme (CASP) tools, depending on study design. Discrepancies were resolved through discussion and consensus. Studies were classified as high, moderate, or low methodological quality based on the proportion of criteria fulfilled.

Although a meta-analysis was initially considered, quantitative synthesis was not conducted because of substantial heterogeneity across studies in terms of intervention types, participant characteristics, outcome measures, and study designs. Consequently, a narrative synthesis approach was adopted to summarize and interpret the findings in accordance with current recommendations for systematic reviews involving heterogeneous evidence.

Because substantial heterogeneity precluded formal statistical subgroup analyses, subgroup comparisons were conducted descriptively through narrative synthesis. Studies were grouped according to intervention type, participant age, and intervention intensity to identify recurring patterns across the evidence base.

The search results presented in Table 1 correspond to the raw number of records retrieved from each database. After combining all records, duplicate references were identified and removed using EndNote and manual verification procedures. Therefore, the number of records reported in the PRISMA flowchart reflects the screening process after database merging and duplicate removal.

All steps of study selection and data extraction were conducted independently by two reviewers, with discrepancies resolved through discussion and consensus. Given the heterogeneity of study designs, interventions, and outcome measures, a quantitative analysis was not feasible. Therefore, the findings were synthesized using a descriptive narrative synthesis approach.

Methodological quality and risk of bias were assessed using the Joanna Briggs Institute (JBI) critical appraisal tools and the Critical Appraisal Skills Programme (CASP) checklists, depending on study design. For randomized controlled trials, domains included randomization, allocation concealment, blinding, outcome measurement validity, and completeness of follow-up. For quasi-experimental and observational studies, additional criteria included confounding control, participant selection, and measurement reliability. Single-case experimental designs were further evaluated using design-specific indicators aligned with What Works Clearinghouse (WWC) standards. Each study was rated as high, moderate, or low quality based on overall criteria fulfillment.

Methodological quality assessment indicated that the overall quality of the included studies was moderate to high. Among the 43 studies assessed using the JBI checklist, 22 were rated as high quality, 17 as moderate quality, and 4 as low quality. Among the 18 studies evaluated using the CASP checklist, 6 were rated as high quality, 8 as moderate quality, and 4 as low quality.

Quality ratings were incorporated into the narrative synthesis and interpretation of findings. Greater interpretive weight was assigned to evidence derived from studies rated as high or moderate quality, whereas findings from low-quality studies were considered supportive but interpreted with caution. The positive effects of digital interventions on receptive and expressive language outcomes were predominantly supported by studies with higher methodological quality. Consequently, the overall conclusions of this review were primarily informed by evidence from studies demonstrating stronger methodological rigor.

Although the review protocol was not formally preregistered, the review procedures were conducted in accordance with PRISMA 2020 reporting guidelines.

### 2.2. Study Selection: Inclusion and Exclusion Criteria

#### 2.2.1. Inclusion Criteria

- Empirical studies published between 2010 and 2025.
- Studies involving children aged 2–18 years diagnosed or identified with Autism Spectrum Disorder (ASD).
- Interventions including digital tools (e.g., mobile applications, tablet-based software, computer-assisted instruction, video modeling, virtual/augmented reality, or artificial intelligence-based adaptive systems).
- Reporting at least one language-related outcome, including receptive language, expressive language, communication attempts, or pragmatic language behaviors (e.g., joint attention).
- Quantitative, quasi-experimental, single-case experimental, or mixed-methods studies reporting measurable outcomes.

#### 2.2.2. Exclusion Criteria

- Articles focusing exclusively on social skills, behavior management, or sensory regulation without language-related outcomes.
- Qualitative studies without quantitative language outcome data.
- Studies involving populations other than children with ASD or mixed samples without separate ASD data.
- Reviews, narrative synthesis, book chapters, theses, technical reports, and conference abstracts.

Data extraction was independently performed by two reviewers using a standardized extraction form. Inter-rater reliability for extracted data demonstrated strong agreement (Cohen’s κ = 0.86; percentage agreement = 93.2%). Any inconsistencies were resolved through consensus-based discussion.

Studies involving participants aged between 2 and 18 years were included to capture the broad developmental variability characterizing language acquisition trajectories in children and adolescents with ASD. This wide age range was considered necessary because digital interventions are implemented across multiple developmental stages, from early language emergence in preschool years to more advanced communicative and pragmatic language skills during adolescence. Although developmental differences across age groups were acknowledged, the review aimed to identify common and age-sensitive patterns regarding the effectiveness of digital language interventions across the ASD population.

This review included experimental, quasi-experimental, and applied observational intervention studies in order to capture the full spectrum of evidence on digital language interventions for children with ASD. Given the relatively emerging and heterogeneous nature of the field, restricting inclusion solely to randomized controlled trials would have excluded a substantial portion of practice-based and ecologically valid intervention research.

Study selection showed substantial agreement (Cohen’s κ = 0.82; percentage agreement = 91.4%). Data extraction demonstrated strong agreement (Cohen’s κ = 0.86; percentage agreement = 93.2%).

Two independent reviewers screened all retrieved records at the title/abstract and full-text stages. Inter-rater agreement was calculated using Cohen’s kappa coefficient and percentage agreement. In cases of disagreement, discrepancies were first discussed between the two reviewers to reach consensus. If consensus could not be achieved, a third senior reviewer acted as an adjudicator and made the final decision. This multi-step procedure was implemented to ensure methodological rigor and minimize selection bias.

Intervention intensity variables, including weekly usage duration and exposure frequency, were extracted only when explicitly reported in the original studies. Due to variability in reporting practices across studies, usage duration findings were synthesized descriptively rather than quantitatively standardized.

A subset of included studies reported effect size estimates, including Cohen’s d, Hedges’ g, and related standardized metrics. These effect sizes were synthesized narratively due to heterogeneity in study designs, outcome measures, and reporting formats. Reported effect sizes generally ranged from small to large across studies, with the majority falling within the small-to-moderate range for receptive and expressive language outcomes.

Stronger effect sizes were more frequently observed in video modeling and structured digital intervention programs, whereas gamified and mobile-based interventions demonstrated more variable effects. Studies reporting higher methodological quality tended to report more stable and interpretable effect size estimates, while lower-quality studies exhibited greater variability.

Given the inconsistent reporting of effect sizes across studies, no formal meta-analytic pooling was conducted; however, all available estimates were systematically extracted and synthesized to support interpretability of findings.

### 2.3. Search Strategy

The search protocol used in this systematic review was predefined and standardized in accordance with PRISMA 2020 reporting guidelines. Search terms were organized around three main concepts: (1) ASD, (2) digital technology-based interventions, and (3) language learning and language development outcomes. Both MeSH terms and free-text keywords were used and adapted to the specific syntax of each database.

This clarification ensures consistent interpretation of outcome measures across included studies and strengthens the internal validity of the review.

Database-specific keyword variations were used to account for differences in indexing systems, controlled vocabularies (e.g., MeSH terms in PubMed), and search engine syntax requirements across databases. Accordingly, search strings were adapted rather than uniformly replicated to maximize sensitivity and specificity within each database. Core conceptual terms (e.g., Autism Spectrum Disorder, digital interventions, and language development) were kept consistent, while synonyms and controlled vocabulary terms were adjusted to align with database-specific indexing structures

This strategy was designed to ensure a balance between sensitivity and specificity in identifying the relevant literature. Table 1 summarizes the search strategy and selection criteria, providing a transparent overview of the review process in line with PRISMA 2020 requirements. The literature search was conducted across multiple electronic databases including PubMed, Scopus, Web of Science, ERIC, and PsycINFO. The search covered studies published between January 2010 and 17 October 2025. The final search was completed on 17 October 2025.

**Figure 1:**
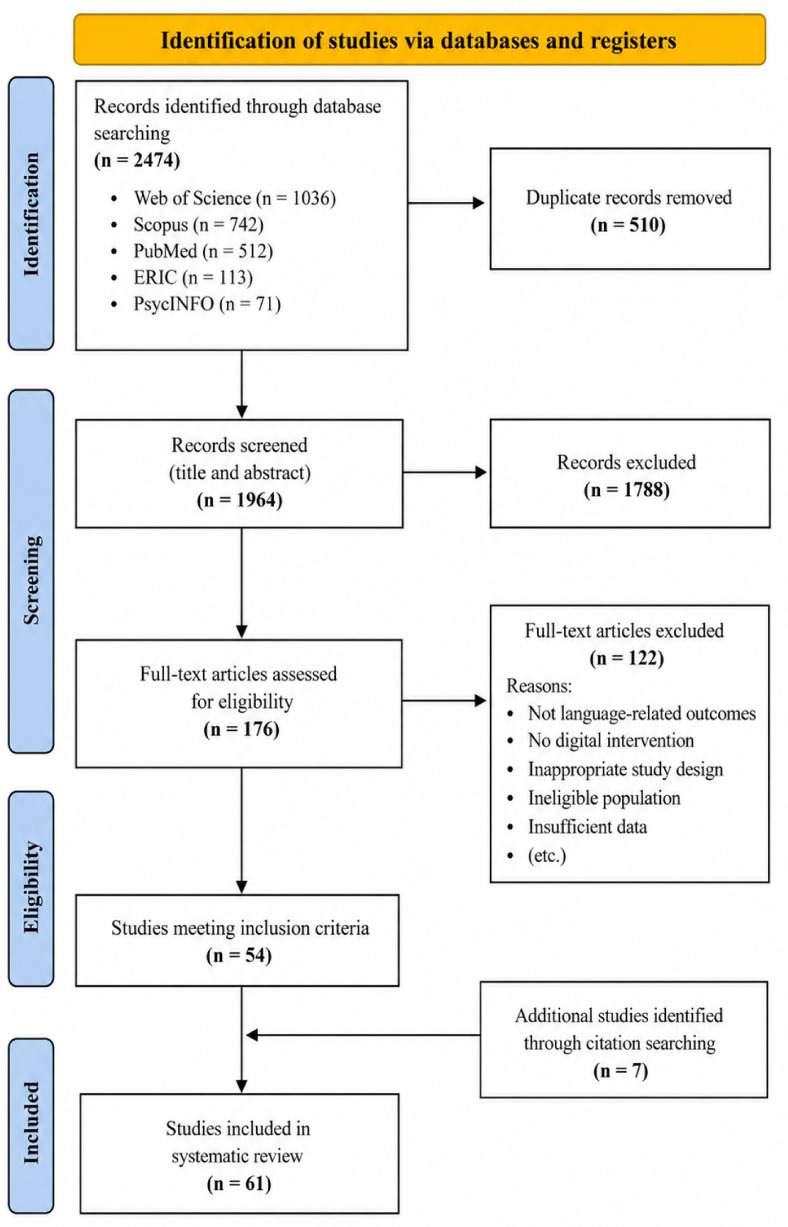
Study selection process and number of studies in the article.

The records reported in this table represent the initial search results obtained from each database before duplicate removal. After merging all records, duplicate citations were removed and the remaining studies were screened according to the PRISMA 2020 procedure presented in Figure 1.

This strategy was designed to ensure a balance between sensitivity and specificity in identifying the relevant literature. Table 1 summarizes the search strategy and selection criteria, providing a transparent overview of the review process in line with PRISMA 2020 requirements. Numbers represent records identified prior to duplicate removal. Duplicates were removed after merging records from all databases, as illustrated in the PRISMA flow diagram. The search procedures yielded a total of 2474 records across the five databases (Table 1). Following the merging of records, 510 duplicate citations were identified and removed, resulting in 1964 unique records for title and abstract screening. After the screening process, 176 articles were retained for full-text assessment. Subsequently, 122 studies were excluded based on the predefined eligibility criteria. An additional seven studies were identified through citation searching, resulting in a final sample of 61 studies included in the review. The complete study selection process is presented in Figure 1 (PRISMA 2020 flow diagram).

The literature search was conducted across multiple electronic databases including PubMed, Web of Science, Scopus, PsycINFO, ERIC. The search strategy combined terms related to Autism Spectrum Disorder, digital interventions, and language development. Boolean operators (AND, OR) were used to refine the search queries according to database-specific requirements. The final search was completed on 17 October 2025.

### 2.4. Data Collection Process

A systematic literature search was conducted in the PubMed, Scopus, Web of Science, PsycINFO, and ERIC databases, with additional supporting searches performed to ensure comprehensive coverage. The search covered studies published between 2010 and March 2025. Database-specific search strings were developed using combinations of keywords related to Autism Spectrum Disorder, digital interventions, and language development, and were adapted to the indexing systems of each database.

The retrieved records were exported to reference management software (e.g., EndNote), where duplicate entries were identified and removed through both automated and manual procedures.

The review process followed the PRISMA 2020 guidelines. Studies were limited to peer-reviewed articles published in English. Review articles, conference proceedings, book chapters, and studies without accessible full texts were excluded.

In the first stage, titles and abstracts were screened based on predefined inclusion and exclusion criteria. Subsequently, the full texts of potentially eligible studies were assessed for final inclusion. The study selection process and the number of records at each stage are presented in Figure 1.

The study selection numbers reported in the PRISMA flow diagram are fully consistent across all stages of identification, screening, eligibility, and inclusion. The retrieved records were exported to reference management software (e.g., EndNote 21), where duplicate entries were identified and removed through both automated and manual procedures.

As illustrated in Figure 1, the study selection process followed a multi-stage procedure in accordance with PRISMA 2020 guidelines. Initially, records were identified through database searches in Web of Science (n = 742), Scopus (n = 1036), and PubMed (n = 512), yielding a total of 2474 records across all databases.

In the identification stage, duplicate records were removed, resulting in 1964 unique studies. During the screening phase, titles and abstracts were evaluated based on predefined inclusion and exclusion criteria, and 1788 records were excluded. Consequently, 176 studies were retained for full-text assessment.

In the eligibility stage, full-text articles were examined in detail, and studies not meeting the inclusion criteria were excluded. This process resulted in 54 eligible studies.

In addition, a backward citation tracking procedure was conducted to enhance the comprehensiveness of the review, leading to the inclusion of 7 additional studies that were not identified through database searches.

In the final stage, a total of 61 studies were included in the systematic review. This stepwise and transparent selection process ensured the derivation of a focused and methodologically robust sample from a large initial pool.

The included studies (n = 61) were conducted across multiple geographic regions, with a predominance of studies from North America, Europe, and East Asia. Most studies included both male and female participants, although a higher proportion of male participants was consistently reported across studies, reflecting the known gender distribution in ASD populations. Baseline language abilities varied widely, ranging from nonverbal children to individuals with functional verbal communication skills

To enhance the transparency and traceability of the screening and selection process, the CADIMA (Computer Aided Design for Systematic Reviews) platform was utilized. This tool facilitated systematic data management, consistency checks, and reviewer agreement procedures. All inclusion and exclusion decisions were documented at each stage, and discrepancies between reviewers were resolved through consensus. The use of this platform strengthened the auditability of the review process and minimized the risk of selection bias.

### 2.5. Protocol Recording and Coding

This systematic review was designed and reported in accordance with the PRISMA 2020 (Preferred Reporting Items for Systematic Reviews) guidelines to ensure transparency, consistency, and reproducibility throughout the review process. These guidelines provide a structured framework for literature identification, study selection, data extraction, quality assessment, and reporting of findings.

The coding framework was developed using a hybrid approach combining deductive and inductive procedures. An initial deductive coding structure was established based on predefined conceptual domains derived from the literature on digital interventions and language development in ASD (e.g., intervention characteristics, language outcome domains, and measurement methods). Subsequently, inductive coding was applied during full-text review to capture emergent variables not fully represented in the initial framework, particularly regarding intervention implementation context and technological variability.

The included studies were categorized based on their research designs into two main groups: experimental studies and observational/applied studies. Experimental studies comprised randomized controlled trials and quasi-experimental designs that evaluated the effects of digital interventions (e.g., tablet-based applications, AI-supported adaptive systems, and video modeling-based instructional approaches) on language development in children with Autism Spectrum Disorder (ASD). Observational/applied studies included research examining the use of digital tools in naturalistic settings or investigating usage patterns and relational outcomes without controlled manipulation.

For each included study, the following variables were systematically coded: publication year, sample size, age range, diagnostic criteria for ASD, intervention duration, type of digital tool, measurement instruments, and reported language outcomes. Language outcomes were classified into widely recognized domains in the literature, including receptive language, expressive language, vocabulary production, pragmatic communication, joint attention behaviors, and communication initiation and maintenance.

Measurement tools used to assess language outcomes were categorized into two main groups based on their methodological characteristics. The first group included standardized instruments with established psychometric properties and normative data, allowing for structured assessment and comparison across age groups. Examples include the Peabody Picture Vocabulary Test for receptive language, Mean Length of Utterance (MLU) for syntactic complexity, and the Clinical Evaluation of Language Fundamentals for comprehensive language evaluation.

The second group consisted of observational and naturalistic assessment approaches, often supported by video recordings. These methods focus on evaluating language use in real-life communication contexts, including indicators such as frequency of communication attempts, joint attention episodes, and initiation of interaction.

Taken together, these two complementary assessment approaches provide a multidimensional evaluation framework that captures both structured language performance and functional language use in natural contexts. This integrated perspective enables a more comprehensive understanding of language development beyond standardized test scores, incorporating the dynamic and contextual aspects of communication. This systematic review was not prospectively registered in any review registry and this review was not registered, and no amendments were made to a publicly available protocol.

### 2.6. Data Analysis and Synthesis Approach

Given the methodological heterogeneity and diversity in language outputs observed in the studies, data analysis was performed in two stages. In the first stage, a comprehensive descriptive and thematic synthesis was carried out. In the second stage, when a sufficient number of homogeneous quantitative studies and comparable statistical data (e.g., mean, standard deviation, sample size) were available, narrative synthesis was performed by calculating effect sizes.

Digital interventions were further classified into subcategories (e.g., AI-supported systems, video modeling interventions, mobile/tablet-based applications, and computer-assisted instructional tools) to improve conceptual consistency during data extraction and narrative synthesis. The synthesis process was guided by developmental, behavioral, and socio-cognitive theoretical perspectives to facilitate interpretation of intervention mechanisms and language-related outcomes across studies.

Study designs were not treated as equivalent in the synthesis process. Instead, findings were interpreted with consideration of methodological strength, with greater interpretive weight given to controlled experimental designs, while applied observational studies were used to contextualize real-world implementation outcomes.

Studies with higher methodological rigor (e.g., controlled experimental designs with clear randomization procedures) were given greater interpretive emphasis, whereas findings from lower-quality studies were interpreted cautiously and used primarily to support contextual understanding.

The final coding framework was refined through iterative comparison across studies, allowing alignment between theory-driven (deductive) categories and data-driven (inductive) refinements.

Given the heterogeneity of included studies, descriptive subgroup comparisons through narrative synthesis based on intervention type (e.g., AI-based systems, video modeling, mobile applications, and computer-assisted instruction), age group (early childhood vs. school-age/adolescence), and intervention intensity (short-, medium-, and long-duration interventions). These subgroup comparisons were used to explore patterns of variation in language outcomes across different intervention characteristics.

To ensure transparency in the interpretation of findings, “consistency levels” (high, moderate, low) were operationally defined prior to synthesis. High consistency was defined as findings showing positive effects in ≥75% of studies within a given category, moderate consistency as 50–74% of studies reporting positive effects, and low consistency as <50% of studies demonstrating positive effects or showing mixed/inconsistent outcomes.

Findings were synthesized according to study design categories, including randomized controlled trials (RCTs), quasi-experimental studies, and single-case experimental designs (SCEDs). These designs were analyzed separately to account for differences in methodological rigor, internal validity, and evidentiary strength. Greater interpretive emphasis was assigned to RCT findings, while quasi-experimental and SCED studies were primarily used to support contextual and exploratory interpretations.

Single-case experimental designs (SCEDs) were appraised using design-specific quality indicators in addition to the JBI and CASP tools. In particular, methodological rigor for SCED studies was evaluated with reference to the What Works Clearinghouse (WWC) standards, including criteria such as independent variable manipulation, inter-observer agreement, repeated measurement, baseline stability, and demonstration of experimental control. SCED studies were categorized as meeting, partially meeting, or not meeting these standards, and this classification informed their weighting in the narrative synthesis. According to the JBI evaluation tool, 43 studies were assessed, of which 22 were classified as high quality, 17 as medium quality, and 4 as low quality. According to the CASP evaluation tool, 18 studies were assessed; of these, 6 were reported as high quality, 8 as medium quality, and 4 as low quality.

## 3. Results

Subgroup analyses indicated that video modeling and AI-based interventions tended to demonstrate more consistent positive effects on both receptive and expressive language outcomes compared to mobile applications and gamified systems. Additionally, interventions with higher intensity and longer duration were generally associated with stronger language gains. Younger children showed relatively stronger improvements in receptive language, whereas older participants demonstrated more variation in pragmatic language outcomes.

Consistency levels were determined using predefined thresholds (high: ≥75%, moderate: 50–74%, low: <50%) based on the proportion of studies reporting positive effects within each category.

Only a subset of included studies reported formal effect size estimates, with values generally indicating small to moderate effects for mobile applications and gamified interventions, and moderate to large effects for video modeling and AI-supported systems. However, variability in reporting practices limited direct comparability across studies.

Methodological quality ratings were integrated into the synthesis to differentiate the weight of evidence across studies. Studies rated as high quality (based on JBI, CASP, and WWC criteria where applicable) were given greater interpretive emphasis in drawing conclusions about intervention effectiveness. Moderate-quality studies contributed to pattern identification, while low-quality studies were interpreted cautiously and primarily used to support contextual understanding rather than effect estimation. Overall, higher-quality studies more consistently demonstrated positive effects of digital interventions on language outcomes in children with ASD.

Reported intervention exposure varied substantially across studies. Several studies described intervention usage ranging approximately from 2 to 5 h per week; however, reporting methods and measurement procedures differed considerably across studies, limiting direct comparability.

Considerable variability was observed across studies regarding participant characteristics and potential confounding variables. Several studies included children with differing ASD severity levels, co-occurring intellectual or developmental conditions, and heterogeneous baseline language profiles. However, reporting of these variables was inconsistent, limiting the ability to systematically compare intervention effectiveness across participant subgroups.

AI-supported interventions constituted approximately 13% of the included studies, limiting the generalizability of conclusions regarding their relative effectiveness.

A Supplementary Table has been added to provide a detailed overview of methodological characteristics across included studies, including design type, sample size, fidelity reporting, outcome measures, follow-up assessment, and risk-of-bias ratings.

A comparative narrative synthesis was conducted across intervention categories, age groups, and intervention intensity levels. Among the included studies, video-modeling interventions most consistently reported positive effects on receptive and expressive language outcomes. AI-supported interventions generally demonstrated favorable results; however, the evidence base was limited to a relatively small number of studies. Gamified and mobile application-based interventions yielded moderate but generally positive effects, particularly for vocabulary acquisition and pragmatic language skills. Across age groups, stronger and more consistent improvements were observed in studies involving younger children, although findings varied according to intervention characteristics and outcome measures. Furthermore, studies implementing interventions over longer durations and with greater intensity tended to report more favorable language outcomes than lower-intensity interventions. These findings represent descriptive patterns identified through narrative synthesis and should not be interpreted as formal statistical subgroup effects.

To enhance transparency, study-level contributions to each synthesis category were explicitly mapped. Video modeling interventions included findings from studies reporting consistent improvements in receptive and expressive language outcomes across multiple experimental and quasi-experimental designs. AI-supported interventions were represented by a smaller subset of studies, which generally reported positive but preliminary effects. Gamified and mobile application-based interventions contributed a broader set of studies showing variable but predominantly positive outcomes, particularly in vocabulary and pragmatic language development.

Across age-related analyses, studies involving younger children predominantly reported stronger language gains, while studies targeting older age groups showed more heterogeneous outcomes. Similarly, high-intensity interventions were primarily supported by studies with longer duration and higher frequency of implementation, whereas lower-intensity interventions contributed mixed findings.

This study-level mapping ensures that each thematic conclusion is directly traceable to the underlying evidence base.

### 3.1. General Characteristics of the Studies

The figure illustrates a gradual increase in the number of publications over time, with a noticeable growth in recent years, indicating the emerging and expanding nature of the field.

**Figure 2:**
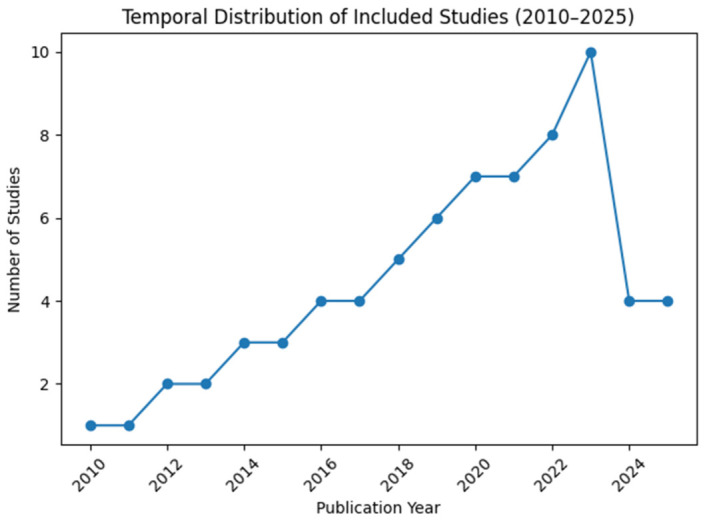
Temporal distribution of studies investigating digital interventions for language development in children with ASD between 2010 and 2025.

Figure 2 presents the temporal distribution of studies examining digital interventions for language development in children with ASD. As shown, there has been a clear increase in publication frequency over time, particularly after 2018, reflecting growing academic interest and technological advancement in this field.

Regarding intervention duration, most studies were conducted within a 1–3 month period (46%), suggesting a predominance of short- to medium-term intervention designs in the literature.

Regarding intervention types, mobile applications (44%) were the most frequently used digital tools, followed by video modeling (23%) and gamified applications (20%). Artificial intelligence and machine learning-based systems were less frequently represented (13%), indicating that these emerging technologies are still relatively underexplored in the field.

Outcome categories were not mutually exclusive, and many studies assessed multiple domains simultaneously. The most commonly evaluated domains were receptive language (57%) and expressive language (52%).

With respect to assessment methods, a relatively balanced distribution was observed between standardized instruments and naturalistic or video-based observations. This finding suggests that the literature integrates both norm-referenced performance measures and ecologically valid observational approaches, enabling a more comprehensive evaluation of language development.

### 3.2. Types and Usage Characteristics of Digital Tools

Within the scope of the analyzed studies, digital interventions were categorized into four main groups based on their primary mode of interaction: mobile applications, game-based systems, video modeling techniques, and AI-supported systems. Each study was assigned to a dominant category according to the primary intervention modality; however, it should be noted that some interventions may incorporate multiple features.

Across the included studies, the average weekly usage time ranged between 2 and 5 h. The highest average usage was observed in game-based systems (approximately 4.0 h per week), whereas video modeling interventions demonstrated comparatively lower usage durations (approximately 2.5 h per week). These estimates were derived from reported intervention protocols and session durations across studies.

In terms of content characteristics, educationally oriented designs were predominant across all categories, accounting for approximately 55–80% of the interventions. The relatively lower representation of entertainment-based and hybrid formats suggests that digital interventions in this field are primarily structured around pedagogical objectives rather than recreational engagement.

Several studies also highlight limitations related to technological infrastructure and device accessibility, particularly in low socioeconomic and rural contexts. These findings indicate that the effectiveness and scalability of digital interventions cannot be fully understood without considering contextual factors. In this regard, access to technology emerges as a potential moderating variable influencing intervention outcomes and equity in implementation.

### 3.3. Language Skills Areas and the Impact of Digital Tools

The included studies examined the effects of digital interventions on multiple domains of language development in children with Autism Spectrum Disorder (ASD). The most frequently assessed domains included language comprehension, expressive language, vocabulary development, pragmatic language use, and language acquisition rate.

The findings summarized in Table 2 and Table 3 were synthesized using a qualitative evidence synthesis approach. The classification of effect levels was based on the consistency, direction, and frequency of reported positive outcomes across studies within each language domain, rather than on reported effects across studies statistical effect sizes. Accordingly, domains in which a majority of studies reported consistent and statistically significant positive outcomes were categorized as “high consistency,” domains with mixed but generally positive findings were categorized as “moderate consistency,” and domains with limited or inconsistent evidence were categorized as “low consistency.”

Video modeling and AI-supported interventions demonstrated high consistency of positive findings, particularly in language comprehension outcomes. The structured, repetitive, and visually supported nature of these interventions appears to facilitate auditory processing and comprehension-related language skills.

Game-based and interactive digital applications were associated with moderate to high consistency in expressive language and vocabulary-related outcomes. These interventions, which emphasize active engagement and learner participation, appear to support productive language use.

Digital games were generally associated with moderate consistency in vocabulary development across both receptive and productive dimensions. In the domain of pragmatic language use, group-based and socially interactive digital activities showed moderate positive consistency, particularly in communication initiation and maintenance behaviors.

Overall, language acquisition rate demonstrated moderate consistency across intervention types; however, this pattern appears to be influenced by intervention duration, intensity of exposure, and individual differences among participants (Table 2).

The synthesis presented in this table is based on findings from the included studies (e.g., video modeling studies; AI-supported intervention studies such as and mobile application-based studies). Intervention effectiveness across language domains reflects aggregated evidence from heterogeneous study designs, including randomized controlled trials, quasi-experimental studies, and single-case experimental designs. Therefore, consistency levels should be interpreted as indicative trends rather than definitive effect estimates.

### 3.4. Measurement Methods

Multiple measurement approaches have been employed in studies to assess language outcomes in children with ASD. Self-report scales, including questionnaires completed by parents, teachers, and in some cases children, have been frequently used to capture perceived language abilities.

Observational methods, including video recordings and direct assessments in structured or semi-structured settings, have been used to examine language performance in more naturalistic or controlled contexts. These approaches provide higher ecological validity, although they may vary in implementation across studies.

Standardized tests have been widely used due to their established reliability and validity in assessing core language domains such as comprehension and production. However, several studies have highlighted the need for ASD-specific adaptations and complementary assessment tools to better capture the heterogeneity of language profiles in this population.

Overall, findings across studies suggest generally positive associations between digital interventions and language outcomes. However, the magnitude and direction of these outcomes appear to vary depending on intervention type, content quality, frequency of use, and socio-demographic characteristics (Table 3).

**Table 3:** Methods used in measuring language skills and their prevalence.

Measurement Method	Definition	Prevalence (%)	Advantages	Limitations
Self-report scales	Questionnaires completed by parents, teachers, and, in some cases, caregivers	55	Practical, easy to administer, captures perceived language abilities	Subject to reporting bias and perceptual limitations
Observational measurements	Video-based analyses, structured or naturalistic observations in controlled settings	30	High ecological validity, captures actual behavioral performance	Time-consuming and resource-intensive
Standardized tests	Validated assessments of language comprehension and production domains	15	High reliability and comparability across studies	May require ASD-specific adaptations to improve sensitivity

Table 3 illustrates the diversity and prevalence of measurement methods used to assess language outcomes in children with ASD. Self-report scales, including questionnaires completed by parents, teachers, and in some cases caregivers, were the most frequently used method in the included studies. Although these instruments are practical and widely applicable, they are subject to reporting bias and may reflect perceptual rather than directly observed abilities.

Observational methods, including video-based analyses and direct observations in structured or semi-structured settings, were used to capture language performance in more naturalistic contexts. These approaches offer higher ecological validity; however, they require substantial time, financial resources, and trained personnel for implementation and analysis.

Standardized tests provide well-established reliability and validity in assessing language comprehension and production, enabling comparability across studies. Nevertheless, several studies highlight the importance of adapting these instruments to better capture the heterogeneous language profiles of children with ASD.

Overall, the integration of multiple measurement approaches contributes to a more comprehensive and multidimensional understanding of language outcomes associated with digital interventions.

Table 3 summarize language outcome domains and measurement approaches across included studies. Across intervention types, digital interventions demonstrated varying degrees of positive impact on language outcomes. Video modeling and AI-supported interventions were most frequently associated with improvements in both receptive and expressive language, with reported positive outcomes in approximately 70–85% of studies. Mobile application-based interventions showed moderate positive effects (approximately 55–70%), particularly in vocabulary development, while gamified interventions demonstrated more variable effects across studies.

Outcome measurements were predominantly conducted using standardized language assessments (e.g., receptive vocabulary tests, expressive language scales), supplemented by observational coding and parent/teacher report measures. Intervention effects were typically assessed immediately post-intervention; however, only a limited number of studies (approximately 20–25%) included follow-up assessments, with follow-up durations ranging from 2 weeks to 6 months. Sustained effects were more consistently reported in video modeling and structured AI-based interventions, whereas mobile and gamified interventions showed less consistent maintenance over time.

### 3.5. Influencing Internal and External Factors

Internal factors influencing the effectiveness of digital tools on language development include cognitive capacity, motivational characteristics, and individual learning-related differences. External factors encompass family support, the quality of the educational environment, and access to digital technologies.

Active parental involvement and guided engagement appear to be associated with more effective use of digital tools and may contribute to more favorable language outcomes. Similarly, the supportive role of educators emerges as an important contextual factor in facilitating learning processes. Conversely, inequalities in access to technology may restrict opportunities for engagement with digital resources, particularly among children from lower socioeconomic backgrounds, and may contribute to widening developmental disparities.

Methodological heterogeneity across studies, as well as variability in sample sizes, should be considered when interpreting the findings, as these factors may limit the broader generalizability of the results (Table 4).

Table 4 summarizes the internal, external, and socio-demographic factors associated with the effectiveness of digital tools on language development in children with ASD. Internal factors include individual characteristics such as cognitive capacity and motivation for learning, which appear to influence the extent to which children engage with and benefit from digital language learning environments.

Percentages reflect the proportion of studies reporting positive language outcomes within each intervention category. Effect duration and follow-up data were inconsistently reported across studies; therefore, long-term effects are based on a limited subset of evidence.

External factors encompass support systems and opportunities provided by the family context, educational settings, and technological infrastructure. Parental involvement in the use of digital tools, teacher guidance in their implementation, and access to adequate devices and internet connectivity are frequently reported as contextual elements associated with children’s digital learning experiences.

Socio-demographic factors, particularly socioeconomic status and geographical location, may influence patterns of access to and engagement with digital tools, potentially contributing to disparities in opportunities for language development. Accordingly, socio-demographic differences should be considered as contextual variables when interpreting the effectiveness of digital interventions (Table 5). A priori coding framework was developed based on the previous literature and refined iteratively during data extraction.

Table 5 outlines the coding framework used to systematically extract and classify data from the included studies. The framework was designed to ensure consistency in categorizing intervention characteristics, language outcome domains, and measurement approaches across studies investigating digital interventions for children with ASD.

The 61 studies included evaluated the impact of digital interventions on language skills in children with ASD, encompassing different research designs, intervention types, and measurement approaches. Findings regarding study characteristics are summarized below (Table 6).

The general characteristics of the included studies are presented in Table 6. The 61 studies included in the review examined the effects of digital interventions on language development in children with ASD, encompassing a range of research designs, intervention modalities, and measurement approaches. The database search initially identified 2474 records. After duplicate removal and eligibility screening procedures, 61 studies met the inclusion criteria and were included in the final synthesis

The majority of studies employed experimental designs (62%), indicating a strong emphasis on evaluating intervention effectiveness within controlled or quasi-experimental frameworks. In terms of sample characteristics, the largest proportion of studies focused on children aged 4–7 years (52%), highlighting early childhood as a primary target period for intervention research.

The temporal distribution of the included studies is presented in Figure 2. The findings indicate a progressive increase in scientific interest in digital interventions targeting language development in children with ASD over the last decade. Early publications between 2010 and 2015 were relatively limited in number, reflecting the emerging nature of the field during this period.

Beyond the increase in publication frequency over time, temporal trends also reflected important methodological and technological shifts within the field. Earlier studies (2010–2015) predominantly focused on computer-assisted instruction and video modeling interventions, often using small-sample or single-case designs. In contrast, studies published after 2018 increasingly incorporated AI-supported systems, mobile applications, and adaptive digital platforms, accompanied by more diverse outcome measures and improved methodological rigor, including greater use of controlled and quasi-experimental designs.

Furthermore, more recent studies tended to report stronger and more consistent effects on receptive and expressive language outcomes, potentially reflecting advancements in intervention personalization, technological interactivity, and implementation quality.

From 2016 onwards, a steady increase in research activity is observed, with a more pronounced growth after 2020. This trend suggests a rapid expansion of interest in technology-assisted language interventions, particularly in response to advancements in mobile applications, artificial intelligence, and digital learning environments.

Overall, the observed distribution highlights that research in this field has transitioned from an exploratory phase to a more established and rapidly developing area of inquiry.

The methodological quality assessment indicated that most included studies demonstrated moderate to high methodological quality. Common strengths included clearly defined intervention procedures, appropriate outcome measures, and detailed participant descriptions. However, several studies showed limitations related to small sample sizes, lack of randomization, insufficient blinding procedures, and limited long-term follow-up. Overall, the risk of bias was considered low to moderate across the included evidence base.

#### 3.5.1. Methodological Quality of Included Studies

The methodological quality assessment using the Joanna Briggs Institute (JBI) checklist and CASP tools indicated variability across included studies. Overall, experimental studies demonstrated moderate to high methodological quality, particularly in terms of outcome measurement and intervention clarity, whereas quasi-experimental and applied observational studies showed more frequent limitations related to sample size, randomization procedures, and blinding. Despite these differences, all included studies were retained in the synthesis due to their relevance to the review objectives. Quality scores were used to inform interpretive weighting rather than exclusion.

#### 3.5.2. Findings by Study Design

Randomized controlled trials generally demonstrated the most consistent evidence regarding the effectiveness of digital interventions on receptive and expressive language outcomes. Quasi-experimental studies showed moderately consistent positive findings, although methodological limitations such as lack of randomization and smaller samples were common. Single-case experimental designs provided detailed evidence regarding individualized intervention responses but demonstrated greater variability in methodological rigor and generalizability.

## 4. Discussion

### 4.1. Principal Findings

The findings of this systematic review demonstrate that the potential of digital interventions to support language development in children with ASD cannot be explained by a one-dimensional “effective/ineffective” dichotomy. The findings of this systematic review demonstrate that digital interventions hold promising potential for language development in children with ASD; however, they necessitate some critical orientations for future research to achieve a higher level of methodological and theoretical maturity in the field. Firstly, future studies should be conducted with larger and more representative samples. Multicenter and cross-cultural designs will increase the generalizability of the findings and comparatively demonstrate the effectiveness of digital interventions in different socio-cultural contexts. Although multiple major databases were systematically searched, the exclusion of non-indexed and difficult-to-retrieve sources may have limited the comprehensiveness of the review. The current literature, dominated by small-sample and single-center studies, offers a limited framework for the stability of effect sizes. Standardization of measurement protocols is necessary. Future research is recommended to develop multi-method designs integrating self-report scales, observational assessments, and standardized tests, and to produce sensitive assessment tools that can capture communication patterns specific to ASD.

The general characteristics of the 61 studies included in this systematic review show that there is a heterogeneous literature, both methodologically and theoretically, when evaluating the effect of digital interventions on the language development of children with ASD. The inclusion of a broad age range increased developmental heterogeneity across studies; however, it also enabled a more comprehensive understanding of how digital interventions may support different stages of language development in children and adolescents with ASD. The inclusion of both experimental and applied observational studies increased methodological heterogeneity; however, it allowed for a more ecologically valid synthesis of digital intervention practices as implemented in real educational and clinical settings.

An important refinement of the present findings concerns the differential contribution of study quality to the overall conclusions. Findings derived from higher-quality studies consistently provide stronger support for the effectiveness of digital interventions in improving receptive and expressive language outcomes in children with ASD. In particular, studies rated as high methodological quality more frequently reported stable and replicable positive effects across intervention types, especially for video modeling and structured digital intervention approaches.

In contrast, evidence from lower-quality studies demonstrated greater variability and, in some cases, less consistent effects. While these studies often reported positive outcomes, their methodological limitations (e.g., small sample sizes, limited control conditions, or incomplete follow-up) necessitate cautious interpretation. Moderate-quality studies generally showed patterns consistent with high-quality evidence but with reduced robustness.

Accordingly, the overall conclusions of this review are primarily driven by higher-quality evidence, while findings from lower-quality studies are considered supportive but not determinative. This distinction enhances the interpretability and methodological transparency of the synthesis.

Overall, the current findings suggest that digital interventions can support language development in children with ASD; however, the effectiveness exhibits a multivariate structure.

A more integrative interpretation of the evidence suggests that the effectiveness of digital interventions in children with ASD is not uniform but systematically moderated by the interaction between intervention characteristics, participant profiles, and implementation context. Specifically, intervention type alone does not fully explain outcome variability; rather, outcomes appear to be shaped by the alignment between intervention structure and developmental needs.

Video modeling and other highly structured digital interventions demonstrate more consistent effects, particularly when applied to younger children and when interventions are delivered with higher intensity. This pattern suggests that structured, repetitive, and visually supported learning environments may better align with the learning profiles commonly observed in ASD.

In contrast, gamified and mobile-based interventions show more variable outcomes, which appear to be influenced by differences in engagement demands and contextual implementation factors such as caregiver involvement and technological accessibility. AI-supported interventions, while promising, remain insufficiently studied to allow robust cross-contextual interpretation, limiting their current explanatory power within the broader evidence base.

Overall, these patterns indicate that intervention effectiveness should be understood as an interactional phenomenon rather than a property of intervention type alone, highlighting the importance of considering developmental stage, intensity, and implementation context simultaneously in future research and practice.

### 4.2. Interpretation in Context

First, the diversity in measurement approaches is noteworthy. While the frequent use of self-report scales is understandable in terms of ease of application, it also brings limitations such as subjective bias and social desirability effects. In contrast, observational assessments and video-based analyses reveal the real-time and contextual use of language skills more objectively; standardized tests, on the other hand, provide comparability and scientific validity, particularly in receptive and expressive language domains. This methodological diversity suggests that the impact of digital tools on language is sensitive to the measurement tool and that the reported impact levels may vary depending on the assessment method. Furthermore, the findings reveal that the effectiveness of the intervention varies not only depending on the type of digital tool used, but also on the quality of the content, frequency of use, and internal characteristics of the child such as cognitive capacity, motivation, and learning speed. In addition, external factors such as family involvement, teacher guidance, and technological access significantly shape the effectiveness of digital interventions [22]. The fact that socioeconomic inequalities, in particular, limit language development opportunities through access to technology demonstrates that the potential of digital interventions cannot be evaluated independently of contextual conditions. In this framework, the impact of digital tools is not linear; it exhibits a multi-layered, interactive, and context-sensitive structure.

The findings obtained in the context of measurement methods parallel ongoing methodological debates in the literature. While the frequent use of self-report scales offers ease of application and low cost advantages, it also carries the risk of subjective bias based on parent and teacher perceptions. Similarly, previous research has shown that parent reports can exhibit optimistic bias, particularly in pragmatic language skills [23,24]. In contrast, observational assessments and analyses based on video recordings more reliably reflect the functional dimension of language use within a natural context. It has been discussed in the literature that laboratory-based direct measurements are sensitive, especially in detecting short-term gains after intervention; however, they have limitations in terms of ecological validity. While standardized tests are known to offer high reliability in language comprehension and production, many studies have highlighted that these tests fail to fully capture the communication patterns specific to ASD and necessitate adaptation [25,26].

Several studies have shown that digital programs offering structured feedback and individualized adaptation report higher effect sizes compared to applications with fixed content [27,28]. Similarly, it has been reported that increasing the weekly intervention duration and total application intensity is a determining factor in language output [29]. However, some studies have indicated that excessive screen time may have negative effects on attention regulation and indirectly limit language acquisition [30]. These conflicting findings suggest that the quality and pedagogical design of digital interventions are more decisive than their quantity.

Compared with previous systematic reviews, the present study offers a broader conceptual integration of heterogeneous digital intervention models and provides a more comprehensive evaluation of language development outcomes in children with ASD. The findings contribute to the literature by synthesizing evidence across multiple technological modalities while also highlighting methodological limitations and contextual moderators that have received comparatively limited attention in earlier reviews.

Although AI-supported systems were associated with potentially favorable language-related outcomes in some studies, they represented a relatively small proportion of the included interventions. Therefore, conclusions regarding the comparative effectiveness of AI-based approaches remain preliminary. The current evidence is insufficient to determine whether AI-supported interventions are consistently superior to more established approaches such as video modeling or computer-assisted instruction.

### 4.3. Methodological Considerations

While the findings of this systematic review are promising, there are some important limitations to consider in interpreting the results. First, there is significant methodological heterogeneity among the included studies. The significant differences in research designs (experimental, quasi-experimental, single-subject designs), sample sizes, and intervention durations have made it difficult to directly compare effect sizes. This limits the generalizability of the findings and causes the synthesis to remain largely descriptive. Second, the diversity in measurement instruments constitutes a significant limitation. The widespread use of self-report scales increases the risk of subjective bias based on parental and teacher perceptions. Although standardized tests offer higher reliability, they may not fully reflect the communication characteristics specific to ASD. The differences in measurement tools may have led to the reported impact levels being sensitive to the measurement method. These limitations do not negate the potential of digital interventions in the language development of children with ASD; however, they clearly demonstrate the need for the field to be strengthened with studies involving larger samples, standardized measurement protocols, long-term follow-up designs, and systematically controlling for contextual variables.

Interpretation of intervention usage duration should be approached cautiously because reporting practices regarding exposure time, adherence, and implementation intensity were inconsistent across the included studies. Consequently, conclusions regarding optimal usage duration remain preliminary. The incomplete and inconsistent reporting of key study-level variables such as intervention fidelity and measurement methods limited the depth of methodological comparison across studies and highlights the need for standardized reporting practices in digital intervention research.

The inclusion of single-case experimental designs introduced additional methodological variability. Although SCEDs provided valuable fine-grained evidence on individual-level intervention effects, not all studies fully met established standards such as WWC criteria, highlighting the need for improved methodological consistency in future SCED research within the ASD digital intervention literature.

The interpretation of intervention effectiveness should be considered in light of several potential confounding variables, including ASD severity, cognitive functioning, comorbidities, and baseline communication abilities. Because many studies reported these variables inconsistently or incompletely, it was difficult to determine the extent to which observed language outcomes were attributable solely to the digital interventions themselves. Future studies should incorporate more standardized participant profiling and statistical control procedures to improve interpretability and comparability across interventions.

Across the included studies, digital interventions were associated with improvements in receptive and expressive language outcomes, particularly in video modeling and AI-supported approaches, although effect sizes and methodological rigor varied considerably.

The limited and inconsistent reporting of effect sizes across included studies represents a key limitation of the current evidence base. This restricts the ability to precisely quantify intervention magnitude and highlights the need for greater statistical standardization in future research.

### 4.4. Implications

The effectiveness of digital interventions is likely influenced by a constellation of contextual factors, including family involvement, socioeconomic conditions, and access to digital technologies. Rather than functioning as isolated therapeutic tools, these interventions operate within broader ecological systems that shape engagement, implementation quality, and learning opportunities. The convergence of these factors may partly explain variability in outcomes across studies.

Developmental differences across the broad participant age range should also be carefully considered when interpreting the findings of this review. Language acquisition processes, cognitive capacities, attention regulation, and social communication demands differ substantially between early childhood, middle childhood, and adolescence in individuals with ASD. Consequently, the effectiveness and appropriateness of digital interventions may vary according to developmental stage and baseline communication profiles.

For example, younger children appeared to benefit more frequently from highly structured and repetitive interventions targeting receptive language and vocabulary acquisition, whereas older children and adolescents demonstrated more variable outcomes related to pragmatic communication, social interaction, and functional language use. These developmental differences may partially explain inconsistencies observed across intervention outcomes and highlight the importance of age-sensitive intervention design and assessment strategies.

The methodological quality assessment revealed substantial variability across included studies, particularly in relation to risk-of-bias domains such as confounding control, blinding, and intervention fidelity. This heterogeneity limits the certainty of conclusions regarding intervention effectiveness and underscores the need for more rigorously designed controlled trials in future research.

The role of socio-demographic and contextual variables is increasingly emphasized in the literature. Studies with high family involvement have reported more pronounced increases in language development; interventions involving parental guidance have been reported to increase generalizability [29]. Teacher-supported interventions have similarly produced more sustainable results [4]. Conversely, it has been noted that limited access to technology in families with low socioeconomic status reduces the intensity of interventions and leads to differentiation in language outputs [11,17]. Previous research also reveals that digital inequality can deepen developmental inequalities [12,15,27,30]. In this context, the impact of digital interventions depends not only on intrinsic factors such as individual cognitive capacity and motivation, but also on external factors such as family support, the quality of the educational environment, and technological infrastructure.

The absence of a quantitative meta-analysis should be considered when interpreting the findings. The considerable methodological and clinical heterogeneity among the included studies precluded the calculation of pooled effect sizes. Future research employing more standardized intervention protocols and outcome measures may facilitate quantitative synthesis and meta-analytic evaluation

Integrating methodological quality into the synthesis revealed that findings from higher-quality studies were more consistent in demonstrating positive effects of digital interventions, whereas lower-quality studies contributed greater variability. This highlights the importance of considering study rigor when interpreting aggregated evidence in heterogeneous intervention research.

Future systematic reviews should further differentiate findings according to methodological rigor and evidentiary hierarchy to improve the precision and reliability of conclusions in this field. Future reviews should incorporate more advanced bias assessment procedures, including publication bias analyses and broader gray literature strategies, to improve the robustness and representativeness of synthesized evidence. Future research should place greater emphasis on developmentally differentiated intervention models and age-specific outcome evaluation frameworks.

## Figures and Tables

**Table 1:** Summary of literature search strategy and selection criteria.

Database	Search History	Search Strategy (Summarized Boolean Structure)	Records Found (n)
PubMed	17 October 2025	(“Autism Spectrum Disorder” OR ASD) AND (digital OR technology-assisted OR mobile application OR tablet application OR video modeling OR virtual reality OR augmented reality OR computer-based training) AND (language development OR communication OR vocabulary OR pragmatic language)	512
Web of Science	17 October 2025	TS = (ASD OR “Autism Spectrum Disorder”) AND TS = (digital OR technology-assisted OR mobile application OR video modeling OR VR OR AR OR computer-based training) AND TS = (language development OR communication OR vocabulary)	1036
Scopus	17 October 2025	TITLE-ABS-KEY (ASD OR “Autism Spectrum Disorder”) AND (digital OR technology-assisted OR mobile application OR video modeling OR virtual reality OR augmented reality) AND (language OR communication OR vocabulary)	742
PsycINFO	17 October 2025	(ASD OR “Autism Spectrum Disorder”) AND (digital intervention OR technology-based OR computer-assisted instruction) AND (communication OR language development)	71
ERIC	17 October 2025	(ASD OR “Autism Spectrum Disorder”) AND (technology-based intervention OR digital learning OR computer-assisted instruction) AND (communication skills OR language development)	113

**Table 2:** The impact of digital tools on the language skills of children with ASD.

Language Skill Domain	Predominant Intervention Types	Consistency Level	Synthesis of Findings
Language comprehension	Video modeling, AI-supported systems	High	Strong and consistent evidence suggests improvements in receptive processing and auditory comprehension skills
Language production	Video modeling, AI-supported systems, interactive applications	Moderate–High	Findings indicate positive effects on expressive language and structured verbal/written output
Vocabulary development	Mobile applications, interactive games	Moderate	Evidence suggests improvements in both receptive and productive vocabulary, with variability across studies
Pragmatic language use	Digital games, group-based digital activities	Moderate	Interventions show positive trends in communication initiation and social language use
Language acquisition rate	Mixed digital interventions	Moderate	Outcomes vary depending on intervention duration, intensity, and participant characteristics

**Table 4:** Factors shaping the impact of digital tools on language development.

Factor Category	Example Factors	Synthesized Interpretation	Supporting Studies	Clarification/Notes
Internal factors	Cognitive capacity, motivation, individual learning-related differences	Individual differences appear to be associated with variability in language outcomes following digital interventions	e.g., [5,17]	Findings primarily derived from mixed-method and quasi-experimental designs; baseline variability not consistently controlled
External factors	Family support, educational environment, access to technology	Parental involvement and educational support are frequently reported as facilitating factors, whereas limited access to technology may constrain opportunities for engagement	e.g., [11,19]	Evidence based largely on observational and implementation-focused studies; intervention fidelity inconsistently reported
Socio-demographic factors	Socioeconomic status, geographical location	Socioeconomic and contextual disparities may influence access to and engagement with digital interventions, potentially contributing to differences in observed outcomes	e.g., [20,21]	Limited number of studies directly tested socio-demographic moderators; findings are correlational in nature

**Table 5:** Coding variables and definitions used in studies examining the impact of digital tools on the language skills of children with ASD.

Main Category	Variable	Subcategory	Definition
I. Intervention Characteristics	Digital tool type	Application/Game-based/Video modeling/AI-supported systems	Type of digital intervention used to support language development
	Intervention duration & intensity	Short/Medium/Long	Total duration or exposure intensity of the intervention
	Content orientation	Educational/Entertainment-based/Hybrid	Degree of pedagogical structuring of digital content
	Social implementation context	Individual/Dyadic/Group-based	Social interaction structure during intervention delivery
	Access to technology	High/Moderate/Limited	Level of availability and usability of required digital resources
II. Language Outcome Domains	Language comprehension	Receptive language/Listening comprehension	Ability to understand and interpret verbal input
	Language production	Spoken language/Written expression	Ability to generate meaningful verbal or written output
	Vocabulary development	Receptive/Expressive vocabulary	Breadth and depth of lexical knowledge
	Pragmatic language use	Communication initiation/Functional use	Ability to use language appropriately in social contexts
III. Measurement Methods	Standardized assessments	Language tests/developmental scales	Norm-referenced or criterion-referenced standardized tools
	Observational methods	Direct observation/Video analysis	Behavioral assessment in naturalistic or structured settings
	Report-based measures	Questionnaires/Interviews	Parent, teacher, or caregiver-reported outcomes

**Table 6:** General characteristics of the included studies (n = 61).

Feature	Category/Values	n	%
Research Design	Experimental (controlled, quasi-experimental)	38	62%
	Observational/Applied	23	38%
Sample Age Range	0–3 years old	15	25%
	4–7 years old	32	52%
	8 years and above	14	23%
Intervention Duration	<1 month	12	20%
	1–3 months	28	46%
	>3 months	21	34%
Type of Digital Tool	Mobile applications	27	44%
	Video modeling	14	23%
	Gamified applications	12	20%
	Artificial intelligence/Machine learning-supported systems	8	13%
Language Skill Outcomes	Receptive language	35	57%
	Expressive language	32	52%
	Vocabulary production	20	33%
	Pragmatic communication	18	30%
	Joint attention behaviors	15	25%
	Communication initiation/maintenance	10	16%
Assessment Tools	Standardized tests (e.g., PPVT, CELF, MLU)	33	54%
	Naturalistic/video-based observations	28	46%

## Data Availability

The data supporting the findings of this systematic review are derived from previously published studies and are publicly available in the databases used for the search, including PubMed, Scopus, Web of Science, and ERIC. All data extracted and analyzed during this study are included in the published article and its Supplementary Materials. No new datasets were generated or analyzed in this study.

## Supplementary Materials

The following supporting information can be downloaded at: https://www.mdpi.com/article/10.3390/pediatric18040090/s1. Table S1: PRISMA 2020 Checklist [31].
